# TLR5-deficiency controls dendritic cell subset development in an autoimmune diabetes-susceptible model

**DOI:** 10.3389/fimmu.2024.1333967

**Published:** 2024-02-28

**Authors:** James Alexander Pearson, Youjia Hu, Jian Peng, F. Susan Wong, Li Wen

**Affiliations:** ^1^ Section of Endocrinology, School of Medicine, Yale University, New Haven, CT, United States; ^2^ Diabetes Research Group, Division of Infection and Immunity, School of Medicine, Cardiff University, Cardiff, United Kingdom

**Keywords:** TLR5, NOD mice, dendritic cells, type 1 diabetes, microbiota

## Abstract

**Introduction:**

The incidence of the autoimmune disease, type 1 diabetes (T1D), has been increasing worldwide and recent studies have shown that the gut microbiota are associated with modulating susceptibility to T1D. Toll-like receptor 5 (TLR5) recognizes bacterial flagellin and is widely expressed on many cells, including dendritic cells (DCs), which are potent antigen-presenting cells (APCs). TLR5 modulates susceptibility to obesity and alters metabolism through gut microbiota; however, little is known about the role TLR5 plays in autoimmunity, especially in T1D.

**Methods:**

To fill this knowledge gap, we generated a TLR5-deficient non-obese diabetic (NOD) mouse, an animal model of human T1D, for study.

**Results:**

We found that TLR5-deficiency led to a reduction in CD11c^+^ DC development *in utero*, prior to microbial colonization, which was maintained into adulthood. This was associated with a bias in the DC populations expressing CD103, with or without CD8α co-expression, and hyper-secretion of different cytokines, both *in vitro* (after stimulation) and directly *ex vivo*. We also found that TLR5-deficient DCs were able to promote polyclonal and islet antigen-specific CD4^+^ T cell proliferation and proinflammatory cytokine secretion. Interestingly, only older TLR5-deficient NOD mice had a greater risk of developing spontaneous T1D compared to wild-type mice.

**Discussion:**

In summary, our data show that TLR5 modulates DC development and enhances cytokine secretion and diabetogenic CD4+ T cell responses. Further investigation into the role of TLR5 in DC development and autoimmune diabetes may give additional insights into the pathogenesis of Type 1 diabetes.

## 1 Introduction

Toll-like receptor 5 (TLR5) is a pattern recognition receptor known to bind bacterial flagellin, a protein found in both Gram-positive and Gram-negative bacteria (1). TLR5 is expressed on different types of cells including dendritic cells (DCs) (2–4), monocytes/macrophages (5), T cells (6, 7), B cells (8), epithelial cells (9–11) and endothelial cells (12). Ligation of TLR5 by flagellin induces downstream signalling via MyD88 and subsequent NF_κ_B activation, leading to the induction of antibody responses by B cells (8, 13, 14) and the induction of Th17 T cell responses (15). All of these have been linked to modified DC development, maturation and function (2–4). Furthermore, TLR5 deficiency has been shown to induce spontaneous colitis (16) and to promote metabolic syndrome in mice, through changing the intestinal microbiota composition (17). A recent study showed that TLR5 expression in intestinal epithelial cells was age-dependent and neonatal TLR5 expression played an important role in shaping the composition of gut microbiota, which had a long-term effect continuing into adulthood (11). Therefore, TLR5 is important in the homeostasis of both immune and metabolic systems.

Type 1 diabetes (T1D), an autoimmune disease, is influenced by both genetic and environmental factors. One of the environmental factors at the interface between the host and the environment is the gut microbiota, which can modulate diabetes risk in humans (18–21). Studies in the Non-obese diabetic (NOD) mouse model, developing spontaneous autoimmune diabetes similar to humans, have also shown that recognition of the microbiota through TLR signalling or Nod-like receptor signalling can modify disease risk (22–30). Therefore, further understanding the interactions between the immune system and gut microbiota is important in developing new strategies to prevent T1D development.

DCs have potent antigen-presenting properties, and induce either inflammation or tolerance, through direct (31, 32) or indirect (33) interaction with the gut microbiota; therefore, DCs can be an important immunotherapeutic target. NOD mice were found to have increased DCs, compared to diabetes-resistant mice (34) and DCs are vital in mediating insulitis and beta cell destruction (35, 36), as without DCs (particularly CD103^+^ DCs), NOD mice do not develop autoimmune diabetes (37). Interestingly, CD103^+^ DCs can sample microbial antigens from the intestinal lumen (31, 33), and it is possible that diabetes protection in the absence of CD103^+^ DCs is related to their microbial antigen sampling function. CD103^+^ intestinal DCs comprise 2 main subsets defined by the presence or absence of CD8α expression; TLR5 is mostly expressed on CD103^+^CD8α^+^ DCs (3). However, little is known of the role of TLR5 in DC development and the role of TLR5 in T1D development.

Here, we report that TLR5-deficiency influences the development of DCs *in utero*, prior to microbial exposure, altering the frequency of different subsets and their responses to stimulation. This effect is maintained to adulthood in the NOD mouse model of spontaneous autoimmune diabetes. In particular, TLR5-deficiency altered both CD103^+^CD8α^+^ and CD103^+^CD8α^-^ DC populations, which coincided with the observed functional changes. TLR5-deficient DCs promoted hyper-secretion of cytokines and proinflammatory responses of both polyclonal and antigen-specific CD4^+^ T cells. Thus, for the first time we report that a TLR-deficiency influences DC development from very early development *in utero*, prior to microbial colonization. However, the effect of TLR5-deficiency in promotion of autoimmune diabetes progression occurs later in life.

## 2 Materials and methods

### 2.1 Mice

NOD/Caj mice have been maintained at Yale University for approximately 30 years. *TLR5^-/-^* C57/BL6 mice were generated as previously reported (1) and the breeding pairs were kindly provided by Richard A. Flavell, Yale University. The strain was backcrossed to our NOD mice for over 10 generations before intercross to otain *TLR5^-/-^* NOD mice. The *TLR5^-/-^* NOD mice used for experiments were obtained from homozoygous breeding (*TLR5^-/-^* NOD x *TLR5^-/-^* NOD or *TLR5^+/+^* NOD x *TLR5^+/+^* NOD). The purity of the NOD genetic background was verified by Illumina mouse whole genome SNP scan (DartMouse™). BDC2.5 and NY8.3 T cell receptor transgenic NOD mice were originally obtained from the Jackson Laboratory and have been bred in-house for over 10 years. All the mice were housed in individually-ventilated filter cages (IVF) in specific pathogen-free (SPF) conditions in a 12-hour dark/light cycle and received autoclaved food ad libitum. For *in utero* experiments, timed matings were set up and monitored daily for plug formation. Seventeen days post-plug, embryos (E17.5) *in utero* were harvested for study. The Institutional Animal Care and Use Committee at Yale University approved the use of mice and the procedures in this study.

### 2.2 Diabetes incidence monitoring

Mice were monitored weekly for diabetes development by testing for glycosuria from 10 weeks of age until the end of the observation period (30 weeks of age). Diabetes was confirmed by two consecutive glycosuria tests, 24 hours apart, with a blood glucose reading of ≥250mg/dl (13.9mmol/L).

### 2.3 Fecal bacterial DNA extraction

Fecal samples from female *TLR5* ^-/-^NOD and *TLR5^+/+^* NOD mice were collected from 8-week-old mice and resuspended in 300µl TE buffer containing 0.5% SDS and 200μg/ml Proteinase K. Bacterial DNA was extracted as previously described (38).

### 2.4 16S rRNA sequencing

The V4 region of the 16S rRNA gene was amplified from each DNA sample using a bar-coded broadly conserved bacterial forward (5’-GTGCCAGCMGCCGCGGTAA-3’) and reverse primer (5’-GGACTACHVGGGTWTCTAAT-3’). PCR products were then purified using a Qiagen gel extraction kit. The DNA concentration was quantified and equimolar amounts of each sample were pooled and used for pyrosequencing with the Ion Torrent PGM sequencing system (Life Technologies). The sequencing data were analyzed with the QIIME software package and UPARSE pipeline to pick operational taxonomic units (OTUs). Taxonomy assignment was performed using representative sequences of each OTU. β-diversity was calculated to compare differences between microbial communities, shown as Principal Coordinate Analysis (PCoA).

### 2.5 Cell surface and intracellular staining for flow cytometry

Cells were harvested from spleen, pancreatic lymph nodes (PLN), mesenteric lymph nodes (MLN) and Peyer’s Patches (PP). 2-5x10^6^ cells were incubated with an Fc blocking antibody (2.4G2; 15 minutes, 4°C) prior to staining with antibodies to CD11b (M1/70), I-A^g7^ (10-3.6, an I-A^k^ antibody cross-reactive to I-A^g7^), CD11c (N418), F4/80 (BM8), CD19 (6D5), TCRβ (H57-597), CD8α (53-6.7), CD103 (2E7), CD4 (GK1.5) and a viability dye for 30 minutes at 4°C. For intracellular staining, cells were stimulated with PMA (50ng/ml, Sigma) and Ionomycin (500ng/ml, Sigma) in the presence of 1μl/ml GolgiPlug™ (BD) for 4 hours. Post-stimulation, cells were stained for the above surface makers. After washing, cells were fixed (20mins at RT) and permeabilized using the eBioscience™ intracellular fixation and permeabilization buffer kit. Cells were then incubated with an Fc blocking antibody (2.4G2; 15 minutes, 4°C) prior to staining for antibodies to IL-4 (11B11), IL-6 (MP5-20F3), IL-10 (JES5-16E3), IL-17A (TC11-18H10.1), granzyme B (GB11), IFNγ (XMG1.2), TNFα (MP6-XT22) and TGFβ (TW7-16B4). FMOs and isotype controls were used for gating. All antibodies were purchased from BioLegend.

### 2.6 Cell isolation

DCs were isolated using the EasySep™ mouse CD11c positive selection kit (Stemcell Technologies) following the manufacturer’s protocol. CD4^+^ T cells were isolated by incubating splenocytes with hybridoma supernatants containing mAbs to CD8 (TB105) and MHCII (10.2.16), while CD8^+^ T cells were isolated by incubating splenocytes with hybridoma supernatants containing mAbs to GK1.5 (CD4) and MHCII (10.2.16), all generously provided by the late Charles Janeway Jr. (Yale University), for 30mins at 4°C. Cells were then washed in PBS and incubated for 45mins on ice with magnetic beads conjugated with goat anti-mouse IgG, goat anti-mouse IgM or goat anti-rat IgG (QIAGEN). CD4^+^ T cells were then negatively isolated using magnetic selection. The purity was routinely 90-95%, as verified by flow cytometry.

### 2.7 qPCR

RNA from purified DCs or small intestinal tissue was extracted using Trizol reagent and RNeasy mini plus kits (QIAGEN). After quantification, 1μg RNA was used for cDNA synthesis using the iScript cDNA synthesis kit (Bio-Rad). Samples were analyzed on an iQ5 qPCR machine (Bio-Rad). Gene expression was determined using the 2^−ΔΔCt^ method and normalized with the housekeeping gene, *GAPDH*. Primers sequences are listed in **Supplementary Table 1**. Each sample was assayed in triplicate and averaged.

### 2.8 Cell culture

Purified DCs (105) were stimulated with one of either PolyIC, CpG, LPS or Pam3Csk4 (all from Invivogen), or anti-CD40 (FGK4.5, BioXcell). CD4^+^ T cells (105) were stimulated with α-CD3 (2C11 hybridoma supernatant), in the presence of mitomycin-c-treated (50µg/ml, Sigma) DCs (105) or stimulated with α-CD3 and α-CD28 (37.51, BioXcell) without DCs for 2 days. For DC co-culture with BDC2.5 CD4^+^ T cells, mitomycin-c-treated DCs (105) were co-cultured with BDC2.5 CD4^+^ T cells in a 1:1 ratio in the presence of BDC2.5 mimotope peptide (RTRPLWVRME) (39). After a 48-hour incubation, the culture supernatants were collected followed by pulsing the cells with ^3^H-thymidine. Cells were incubated for an additional 18 hours before harvesting. Proliferation was determined by ^3^H-Thymidine incorporation using a β-counter and are presented as counts per minute (CPM), after subtracting background (cells without stimulation), termed ΔCPM.

### 2.9 *In vivo* cell transfer

BDC2.5 CD4^+^ T cells were isolated as above, prior to labelling with CFDA-SE (5μM) for 15mins at 37°C. 2x10^6^ CFSE-labelled cells were iv transferred into 8-week-old *TLR5* ^-/-^NOD and *TLR5^+/+^* NOD recipients. *In vivo* proliferation (dilution of CFSE) was assessed 3 days post-injection from the PLN and islets by flow cyotmetry.

### 2.10 Microbe-splenocyte co-culture

Fecal pellets were harvested from 2-month-old female *TLR5* ^−/−^NOD and *TLR5* ^+/+^NOD mice and homogenized in sterile PBS using a bead beater machine (BioSpec). Fecal material was then centrifuged for 2 mins at low speed (52 × g) to remove dietary residue. The supernatant was transferred to a new tube and spun. After washing the pellet two more times, the combined supernatant was centrifuged at 469 × g to remove mammalian cells. Bacteria in the supernatant were pelleted by centrifugation at high speed (1876 × g, 5 min) and resuspended in sterile PBS. Bacterial concentration was measured with a spectrophotometer (Bio-Rad) and heat-inactivated at 90°C for 20 min. The heat-inactivated bacteria (108) were co-cultured with splenocytes (2 × 10^6^/ml) from *TLR5* ^−/−^NOD or *TLR5* ^+/+^NOD mice for 12 hours prior to intracellular cytokine (ICC) staining.

### 2.11 ELISA

Cytokines were detected in cell culture supernatants at 48 hours after stimulation. TGFβ was detected using a mouse TGFβ1 DuoSet ELISA kit (R&D Systems), while IFNα was detected using a Verikine Mouse IFNα Elisa Kit (PBL Assay Science). All other cytokines were detected using anti-mouse cytokine ELISAs from BioLegend. All were conducted following the manufacturer’s protocols.

### 2.12 Statistics

Data with a larger sample size were assessed for normality using a D’Agostino & Pearson test (p<0.05 identified any data not normally distributed), prior to a Student’s T Test being performed to compare WT and KO data for those that were normally distributed. In some cases, due to small sample size (n=4) the D’Agostino & Pearson test was unable to determine whether the data were normally distributed. Dependent on sample size, a Mann-whitney (n=3-4) or two-way ANOVA (n=8-9) analysis were performed for *in vitro* proliferation and measurements of secreted cytokines in order to compare WT and KO mice.

## 3 Results

### 3.1 TLR5 expression influences dendritic cell development, prior to microbial colonization, *in utero*

**Figure 1:**
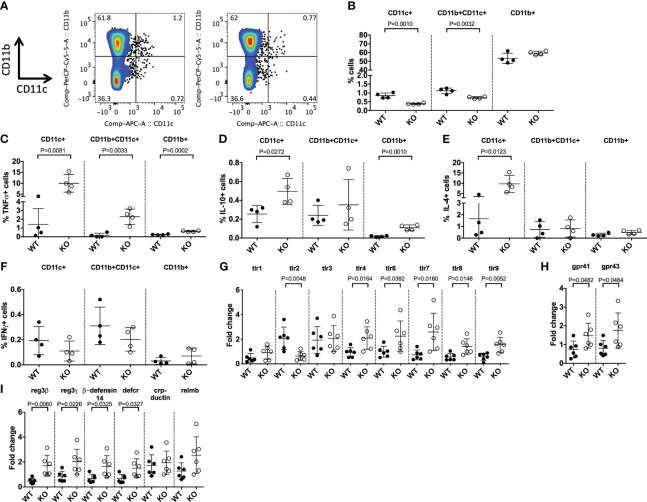
TLR5-deficiency alters DCs *in utero*. Splenocytes from E17 mice *in utero* were harvested from TLR5-sufficient (WT) or TLR5-deficient (KO) pregnant females, 17-days post-plug formation, prior to cell surface **(A, B)** or intracellular staining **(C–F)**. **(A)** Representative CD11b vs CD11c flow cytometric plots gated on single, live CD19^-^CD3^-^ cells. **(B)** Summarized data from **(A)**. **(C–F)** Cytokine-producing splenic CD11b^-^CD11c^+^ (designated CD11c^+^), CD11b^+^CD11c^+^ or CD11b^+^CD11c^-^ (designated CD11b^+^) cells were gated as the cells in **(A)** prior to gating on TNFα^+^ **(C)**, IL-10^+^ **(D)**, IL-4^+^ **(E)** or IFNγ^+^ populations. **(G–I)** Intestinal tissue from the mice *in utero* were snap frozen, prior to RNA extraction and qPCR for TLRs **(G)**, SCFA receptors **(H)** or antimicrobial peptides **(I)**. Samples were averaged from triplicates with the relative gene expression determined using the 2^-ΔΔCT^ method by normalization with the housekeeping gene, GAPDH. Data shown are from 1 of 2 representative independent experiments (**A-I**: n=4/experiment) and were assessed for significance using a Student’s T Test.

Age-dependent expression of TLR5 has been reported in intestinal epithelial cells from neonatal mice (11); however the role of TLR5 in early intestinal and the immune cell development *in utero* is completely unknown. We first investigated whether TLR5 expression influenced the development of immune cells *in utero* by studying *TLR5^+/+^* (WT) NOD mice and *TLR5^-/-^* (KO) NOD mice at E17.5 days. Interestingly, we found a significant reduction in the proportion of both splenic CD11b^-^CD11c^+^ and CD11b^+^CD11c^+^ DCs from developing KO mice compared to WT controls (**Figures 1A, B**, **Supplementary Figure 1**). This proportional change in DCs was also associated with increased TNFα, IL-10 and IL-4 cytokine secretion from the cells of the KO mice, while no changes were found in IFNγ secretion from these cells (**Figures 1C–F**, **Supplementary Figures 2**-**4**). Investigation of adaptive immune cells revealed no proportional changes in splenic B cells or in their secretion of cytokines (**Supplementary Figures 5A, B**) and no changes in the frequency of thymocytes or peripheral splenic T cells (**Supplementary Figures 5C, D**). Therefore, TLR5-deficiency predominantly altered the development of DCs early in life, prior to microbial colonization. To determine the impact of TLR5-deficiency on prenatal intestinal immunity we investigated changes in the expression of microbial response genes from the intestine of the E17.5 mice. We found that TLR5-deficiency increased the gene expression of most TLRs, with the exception of *tlr2* (reduced) and *tlr1* and *tlr3* (unchanged) (**Figure 1G**). We also found that the expression of the receptors for microbe-derived short-chain fatty acids (SCFAs) (gpr41 and gpr43) (40) were both increased in the intestine of prenatal TLR5 KO mice compared to WT controls (**Figure 1H**). Investigation of intestinal antimicrobial peptides also revealed that 4 out of 6 antimicrobial peptides tested were increased in TLR5 KO mice vs WT controls (**Figure 1I**). It has been reported that human fetal development does not occur in sterile conditions, as bacteria have been detected in the intrauterine environment (41–44); however, in our prenatal mice we found, by 16S qPCR, no evidence of microbial colonization (data not shown). Together, these data suggested that TLR5 affects DC development and intestinal anti-microbial immunity *in utero*, prior to microbial colonization.

### 3.2 TLR5 expression influences DCs in adult mice

**Figure 2:**
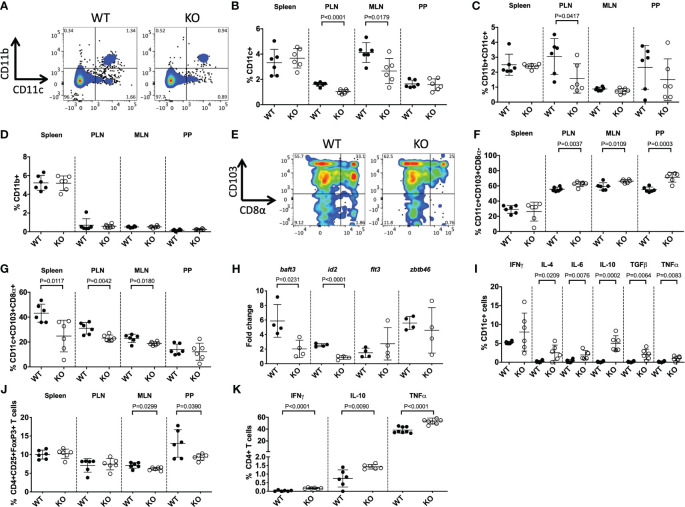
TLR5-deficiency alters DC subsets in adult mice. DC subsets from 8-week-old TLR5-sufficient (WT) or TLR5-deficient (KO) mice were studied. **(A–D)** Representative flow cytometric gating **(A)** and the proportion of CD11b^-^CD11c^+^ (designated CD11c^+^) **(B)**, CD11b^+^CD11c^+^ **(C)** and CD11b^+^ CD11b^+^CD11c^-^ (designated CD11b^+^) **(D)** cells investigated from the spleen, pancreatic lymph nodes (PLN), mesenteric lymph nodes (MLN) and Peyer’s patches (PP). Cells were gated from live, single CD19^-^TCRβ^-^ cells. **(E–G)** Representative flow cytometric gating **(E)** and the proportion of CD11c^+^CD103^+^CD8α^-^ **(F)** and CD11c^+^CD103^+^CD8α^+^ DCs **(G)**. Cells were gated as above, prior to gating on CD103 and CD8α. **(H)** Purified splenic CD11b^-^CD11c^+^ DCs were snap frozen, prior to RNA extraction and qPCR for genes associated with DC development. Samples were averaged from triplicates, with the relative gene expression determined using the 2^-ΔΔCT^ method by normalization with the housekeeping gene, GAPDH. **(I)** The proportion of cytokine-secreting DCs, from the PLN, were investigated by flow cytometry. Cells were gated as in **(A)**, prior to gating on IFNγ^+^, IL-4^+^, IL-6^+^, IL-10^+^, TGFβ^+^ or TNFα^+^ populations. **(J)** The proportion of Tregs, gated from live, single TCRβ^+^CD4^+^CD19^-^CD8^-^ cells, prior to gating on CD25^+^FoxP3^+^ cells. **(K)** The proportion of cytokine-secreting CD4^+^ T cells, from the PLN, were investigated by flow cytometry. Cells were gated from live, single TCRβ^+^CD4^+^CD19^-^CD8^-^ cells, prior to gating on IFNγ^+^, IL-10^+^ or TNFα^+^ populations. Data shown are pooled from 2 independent experiments (**A-K**: n=4-6/experiment) and were assessed for significance using a Student’s T Test.

Having observed suppressed CD11c^+^ DC development in prenatal TLR5-deficient mice, we asked if this was specifically related to the TLR5 deficiency or was an age-dependent observation. We assessed sexually mature 8-week-old adult mice. We found that both CD11b^-^CD11c^+^ and CD11b^+^CD11c^+^ DCs were reduced in TLR5 KO mice in the pancreatic lymph nodes (PLN; draining lymph nodes for the pancreas) and CD11b^-^CD11c^+^ DCs were reduced in the mesenteric lymph nodes (MLN), compared to WT mice (**Figures 2A–C**, **Supplementary Figure 6**). Similar to the prenatal mice, there were no differences in the proportion of CD11b^+^ cells (**Figure 2D**). We further investigated the DC subsets, and found that TLR5 deficiency was associated with increases in the migratory CD103^+^CD8α^-^ DCs, in the PLN, MLN and the Peyer’s patches (PP) (**Figures 2E, F**) of KO mice when compared to WT NOD mice. Along with the increase of CD103^+^CD8α^-^ DCs, there was a decrease of the CD103^+^CD8α^+^ DC subset in KO mice (**Figure 2G**). The CD103^+^CD8α^-^ and CD103^+^CD8α^+^ subsets were both unaltered in CD11b+CD11c^+^ DCs and CD11b^+^CD11c^-^ macrophages (**Supplementary Figures 7A–D**). Gene expression analysis of CD11c^+^ DCs by qPCR revealed that genes involved in DC development, *baft3* and *id2*, were significantly decreased, while no differences were seen in *flt3* and *zbtb46* expression between WT and KO mice (**Figure 2H**). Interestingly, Baft3 and Id2 are important for the development of CD8α^+^ DCs (45, 46), which are reduced in the TLR5 KO mice. Similar to the prenatal mice, we also observed increased cytokine secretion, except IFNγ, from CD11c^+^ DCs in the PLN of the TLR5 KO mice (**Figure 2I**, **Supplementary Figure 8**). The changes in cytokines, mostly IL-6, TGFβ and TNFα, were also found in CD11b^+^CD11c^+^ DCs and CD11b^+^CD11c^-^ macrophages in TLR5 KO mice (**Supplementary Figures 7E, F**). As DCs are intimately in contact with B cells and T cells, we then investigated these adaptive immune cells. We found that in the absence of TLR5, B cells also had altered cytokine secretion, as well as the proportion of CD138^+^ plasma cells and IgA^+^ B cells (**Supplementary Figures 7G–I**). Interestingly, in the absence of TLR5, the proportion of regulatory T cells in the MLN and PP was reduced but cytokine secretion by CD4^+^ T cells, but not CD8^+^ T cells, was increased in the PLN (**Figures 2J-K**, **Supplementary Figures 7J**, **9**).

### 3.3 TLR5 modulates the function of DCs

**Figure 3:**
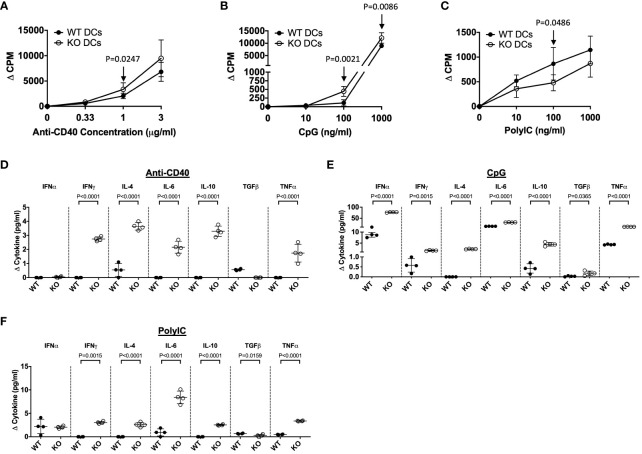
TLR5-deficiency alters DC proliferation to immune stimuli but increases cytokine production. CD11c^+^ DCs were purified from the spleen of 8-week-old TLR5-sufficient (WT) or TLR5-deficient (KO) mice and stimulated for 48 hours in the presence of various concentrations of anti-CD40 **(A)**, CpG (**B**; TLR9 agonist) or PolyIC (**C**; TLR3 agonist), prior to supernatant collection and the addition of ^3^H-thymidine. Cells were incubated for a further 18 hours prior to harvesting and analysis on a β-counter. **(D–F)** Cell supernatants from the DC stimulations **(A–C)** were investigated for cytokine concentrations by ELISA. Data shown are from the highest concentration of stimulation. Data are plotted as counts per minute, corrected for background (no stimulation (ΔCPM)) or cytokine concentration, corrected for background (no stimulation (Δ Cytokine (pg/ml))). Data are averaged from triplicates or duplicates respectively. Data shown are representative of 1 of 3 independent experiments (**A–F**: n=3-4/experiment) and were assessed for significance using a Mann Whitney analysis.

As TLR5-deficiency substantially alters DC development, we investigated DC responses to innate and adaptive immune stimuli, to identify if these functional responses were also affected by the absence of TLR5 in adult. Purified splenic CD11c^+^ DCs from TLR5-sufficient and -deficient NOD mice (8-weeks old) were stimulated with TLR ligands (innate stimuli) or anti-CD40 (adaptive stimulus). The innate stimuli used in the assay were Pam3Csk4 (for TLR2), PolyIC (for TLR3), LPS (for TLR4) and CpG (for TLR9). Interestingly, we found that TLR5-deficient DCs showed increased proliferative responses to CpG and anti-CD40 stimulation but reduced proliferative response to PolyIC (**Figures 3A–C**). It appeared that the presence or the absence of TLR5 did not affect the DC proliferative response to Pam3Csk4 or LPS (**Supplementary Figures 10A, B**). As we identified increased cytokine responses to mitogen stimulation, by the TLR5-deficient DCs from both prenatal and adult mice, we next determined whether cytokine secretion was also altered in response to the innate and adaptive immune stimulation *in vitro*. It is intriguing that all the cytokines tested (IFNγ, IL-4, IL-6, IL-10 and TNFα) were highly increased in the cultures with the TLR5-deficient DCs in response to different stimuli (**Figures 3D–F**, **Supplementary Figures 10C, D**). However, the increase in IFNα and TGFβ secretion by TLR5-deficient DCs was only found with CpG stimulation (**Figure 3E**). Interestingly, TGFβ secretion was reduced in TLR5-deficient DCs in response to PolyIC and LPS stimulation when compared to TLR5-sufficient DCs (**Figure 3F**, **Supplementary Figure 10C**). Together, these data demonstrate that TLR5-deficiency also alters the function of DCs in response to both innate and adaptive immune stimuli, compared to TLR5-sufficient DCs.

### 3.4 Gut microbiota from TLR5-deficient mice directly modulate cytokine production by immune cells

**Figure 4:**
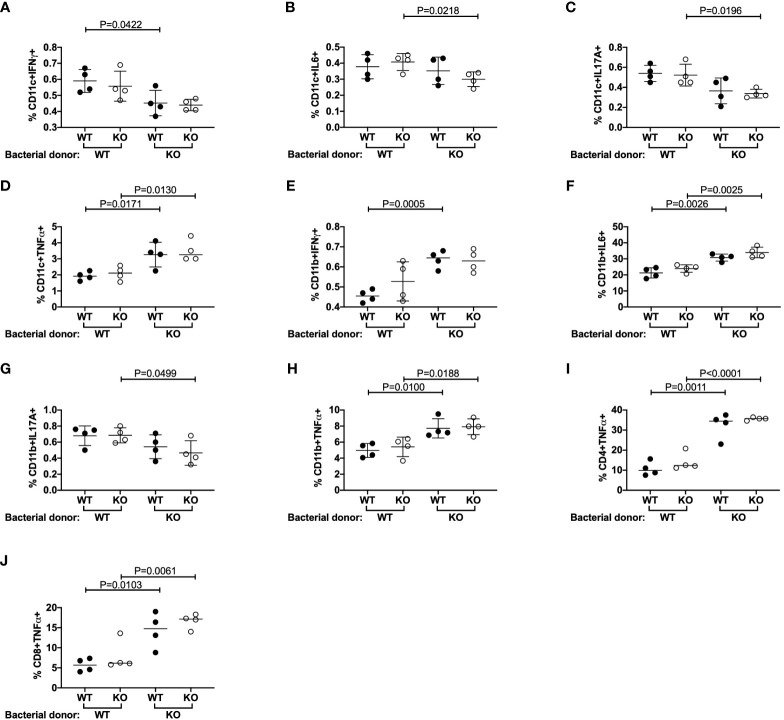
Microbiota from TLR5-deficient mice alter immune cell secreted cytokines. Splenocytes and fecal microbiota from 8-week-old TLR5-sufficient (WT) and TLR5-deficient (KO) mice were harvested. Fecal microbiota were isolated and heat-inactivated prior to culture with splenocytes overnight. 12 hours later, cells were stimulated for 4 hours with PMA and Ionomycin in the presence of GolgiPlug prior to cell surface and intracellular staining. CD11c^+^ DCs **(A–D)** and CD11b^+^ macrophages **(E–H)** were investigated for their proportion of IFNγ^+^, IL-6^+^, IL-17A^+^ or TNFα^+^ cells respectively, by flow cytometry. CD11c^+^ DCs and CD11b^+^ macrophages were gated from live, single I-Ag7^+^CD19^-^TCRβ^-^ cells, prior to gating on CD11c^+^CD11b^-^ or CD11b^+^CD11c^-^ populations respectively. **(I, J)** CD4^+^ and CD8^+^ T cells were investigated for their proportion of TNFα^+^ T cells by flow cytometry. T cells were gated from live, single CD19^-^TCRβ^+^ cells, prior to gating on CD4^+^CD8^-^ or CD8^+^CD4^-^ populations respectively. WT and KO labels on the x axis refer to splenocyte donor and are grouped to indicate the bacterial donor. Data shown are representative of 1 of 3 independent experiments (**A–J**: n=4-5/experiment) and were assessed for significance using a Student’s T Test.

Previous studies reported that TLR5-deficient C57BL/6 mice had altered microbiota (11, 17). We did not find noticeable differences in α-diversity, a measure of the number of microbiota present, between our TLR5-deficient and TLR5-sufficient NOD mice (data not shown); however, we did observe significant differences in β-diversity, a measure of the types of microbiota present (**Supplementary Figure 11A**). The differences were mostly observed in the proportion of 4 microbial species (**Supplementary Figures 11B–E**). This confirmed the previous findings that TLR5 influences the composition of gut microbiota. As our data suggested that TLR5-deficient DCs were hyper cytokine-producers in response to TLR ligand stimulation, we hypothesized that altered gut microbiota in TLR5-deficient NOD mice directly affect the function of DCs. To test our hypothesis, we stimulated the splenocytes from TLR5-sufficient or –deficient NOD mice with heat-inactivated fecal microbiota, collected from either TLR5-sufficient or -deficient NOD mice in a criss-cross manner. Interestingly, we found that gut bacteria from the TLR5-deficient mice suppressed secretion of IFNγ, IL-6 and IL17A but promoted TNFα secretion, in both TLR5-sufficient and -deficient DCs, compared to microbiota from TLR5-sufficient NOD mice (**Figures 4A–D**, **Supplementary Figure 12**). However, CD11b^+^CD11c^-^ macrophages increased production of TNFα-, IFNγ- and IL-6 but decreased IL17A-secretion in response to microbiota from TLR5-deficient mice (**Figures 4E-H**, **Supplementary Figure 13**). Moreover, microbiota from TLR5-deficient mice promoted more TNFα-producing CD4^+^ and CD8^+^ T cells, compared to the microbiota from TLR5-sufficient mice (**Figures 4I, J**, **Supplementary Figure 14**), whereas cytokine production from B cells did not appear to be affected (data not shown). Taken together, our results suggest that the microbiota from TLR5-deficient mice can directly modulate cytokine secretion by a number of different immune cells.

### 3.5 TLR5-deficient DCs modulate CD4^+^ T cell responses

**Figure 5:**
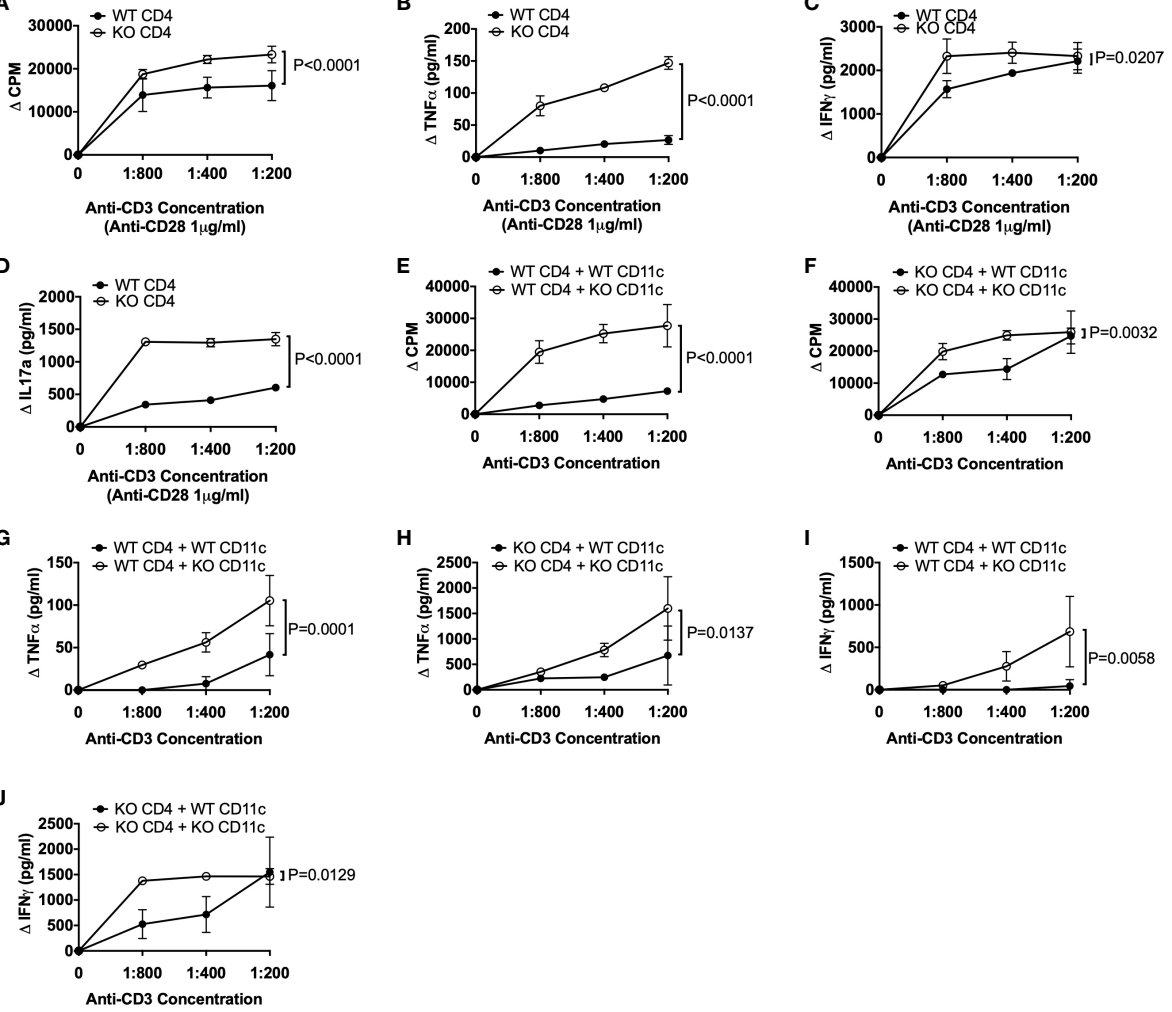
TLR5-deficient CD4^+^ T cells are more inflammatory and modulated by TLR5-deficient DCs. **(A–D)** Splenic CD4^+^ T cells were purified from 8-week-old TLR5-sufficient (WT) and TLR5-deficient (KO) mice. CD4^+^ T cells (2x10^5^/well) were cultured in the presence of various concentrations of anti-CD3 with 1μg/ml anti-CD28 for 48 hours, prior to supernatant collection and ^3^H-Thymidine addition. Cells were cultured for a further 18 hours prior to harvesting. Proliferation of CD4^+^ T cells **(A)** was analyzed on a β-counter. Secreted TNFα **(B)**, IFNγ **(C)** and IL-17A **(D)** cytokines were measured from the supernatant of cell cultures in **(A)**. **(E–J)** Splenic CD4^+^ T cells and CD11c^+^ DCs were purified from 8-week-old TLR5-sufficient (WT) and TLR5-deficient (KO) mice. CD4^+^ T cells (1x10^5^/well) and mitomycin-c-treated CD11c^+^ DCs (1x10^5^/well) were cultured 1:1 in the presence of various concentrations of anti-CD3 for 48 hours prior to supernatant collection and ^3^H-Thymidine addition. Cells were cultured for a further 18 hours prior to harvesting. ^3^H-Thymidine incorporation proliferation of WT CD4^+^ T cells in the presence of WT or KO CD11c^+^ DCs **(E)** or KO CD4^+^ T cells in the presence of WT or KO CD11c^+^ DCs **(F)** was analyzed on a β-counter. Secreted TNFα **(G, H)** and IFNγ **(I, J)** cytokines were measured from the supernatants in **(E, F)**. Data shown are pooled from 3 independent experiments (**A–J**: n=8, n=2-3/experiment) and were assessed for significance using a two-way ANOVA.

**Figure 6:**
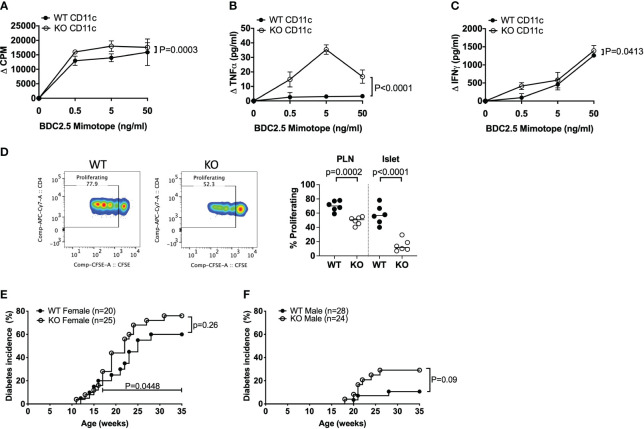
TLR5-deficient DCs modulate antigen-specific CD4^+^ T cells and increase diabetes development in older mice. **(A–C)** CD11c^+^ DCs were purified from 8-week-old TLR5-sufficient (WT) and TLR5-deficient (KO) mice. CD4^+^ T cells were purified from BDC2.5 T cell receptor transgenic mice. BDC2.5 CD4^+^ T cells (1x10^5^/well) and mitomycin-c-treated CD11c^+^ DCs (1x10^5^/well) were cultured 1:1 in the presence of various concentrations of BDC mimotope peptide for 48 hours prior to supernatant collection and ^3^H-Thymidine addition. Cells were cultured for a further 18 hours prior to harvesting. ^3^H-Thymidine incorporation proliferation of BDC2.5 CD4^+^ T cells in the presence of WT or KO CD11c^+^ was analyzed on a β-counter. Secreted TNFα **(B)** and IFNγ **(C)** cytokines were measured from the supernatants in **(A)**. Data shown are pooled from 3 independent experiments (n=9, n=3 per experiment). **(D)** CFSE-labelled BDC2.5 CD4^+^ T cells (2x10^6^) were adoptively transferred into WT or KO mice and assessed for proliferation after 3 days *in vivo*. Cells were gated on live, single CD4^+^CD8^-^ cells prior to gating on CFSE^+^ cells and subsequently those cells proliferating (dilution of CFSE). Data shown are pooled from 2 experiments (n=6, n=3 per experiment). **(E, F)** TLR5-sufficient (WT) and TLR5-deficient (KO) female **(D)** and male **(E)** mice were assessed weekly for spontaneous diabetes development by glycosuria, with diabetes confirmed with a blood glucose >250mg/dl. Data in D was assessed for significance for both the overall diabetes incidence (p=0.26) and the incidence of diabetes from 17 weeks of age to termination (p=0.0448). Data were assessed for significance using a two-way ANOVA **(A–C)**, Student’s T-test **(D)** or a Gehan-Breslow-Wilcoxon test **(E, F)**.

As CD4^+^ T cells can express TLR5 (6, 7) and we found increased TNFα-secreting CD4^+^ T cells in TLR5-deficient NOD mice both ex-vivo (**Figure 2K**) and in response to the gut microbiota from TLR5-deficient NOD mice (**Figure 4I**), we asked whether TLR5-deficiency has a direct effect on CD4^+^ T cells or the observed changes in CD4^+^ T cells occur through DCs. To test this hypothesis, we cultured the purified CD4^+^ T cells, from TLR5-sufficient and TLR5-deficient NOD mice, in the presence of anti-CD3 and anti-CD28. CD4^+^ T cells from TLR5-deficient mice not only proliferated more, but also secreted higher levels of TNFα, IFNγ and IL17A, in response to TCR activation, than CD4^+^ T cells from TLR5-sufficient mice (**Figures 5A–D**). As anti-CD28 provided the co-stimulation as 2^nd^ signal, for TCR activation, we asked whether the enhanced response by TLR5-deficient CD4^+^ T cells was attributable to DCs that are not only potent antigen presenting cells providing co-stimulation to T cells but also are altered in the absence of TLR5 (**Figures 1**–**3**). To address this, we purified DCs from TLR5-sufficient and TLR5-deficient mice and co-cultured them with either TLR5-sufficient or TLR5-deficient CD4^+^ T cells in a criss-cross manner (**Figures 5E, F**). We showed that, compared to the DCs from TLR5-sufficient mice, DCs from TLR5-deficient mice were able to promote stronger proliferation of both TLR5-sufficient and TLR5-deficient CD4^+^ T cells (**Figures 5E, F**). In addition, TNFα and IFNγ secretion was increased when CD4^+^ T cells (regardless of which donor mice were used) were co-cultured with DCs from TLR5-deficient mice (**Figures 5G–J**). This suggested that the enhanced responses by TLR5-deficient CD4^+^ T cells were most likely to be mediated by TLR5-deficient DCs. To determine if this also applied to antigen-specific CD4^+^ T cells, we co-cultured DCs with diabetogenic BDC2.5 CD4^+^ T cells in the presence of antigenic peptide. Similar to the polyclonal CD4^+^ T cell response, TLR5-deficient DCs enhanced the proliferation of islet antigen-specific BDC2.5 CD4^+^ T cells compared to the TLR5-sufficient DCs (**Figure 6A**). As observed for the polyclonal CD4^+^ T cells (**Figure 5**), the TLR5-deficient DCs promoted a stronger proinflammatory TNFα and IFNγ secretory response from oligoclonal CD4^+^ T cells, compared to the TLR5-sufficient DCs (**Figures 6B, C**). We did not observe any differences in the ability of DCs to present antigen to IGRP-specific CD8^+^ T cells from NY8.3 NOD mice *in vitro* (**Supplementary Figure 15**), suggesting that DCs more strongly affected CD4^+^ T cell activation. To assess *in vivo* proliferation of BDC2.5 CD4^+^ T cells in TLR5-sufficient and -deficient hosts, CFSE-labelled BDC2.5 CD4^+^ T cells were intravenously transferred into WT and TLR5-deficient mice, prior to determination of proliferation, 3 days later. Similarly, we found that BDC2.5 T cells proliferated less in TLR5-deficient hosts than TLR5-sufficient hosts in both the PLN and islets (**Figure 6D**, **Supplementary Figure 16**). Finally, to determine whether all the changes in the absence of TLR5 altered T1D susceptibility, we observed the spontaneous autoimmune diabetes development in TLR5-sufficient and TLR5-deficient mice. TLR5-deficient NOD mice developed a higher incidence of T1D than the TLR5-sufficient NOD mice, although the difference did not reach statistical significance (**Figures 6E, F**). Interestingly, analysis of the incidence of older female mice (over 17 weeks of age), revealed that TLR5-deficient NOD mice developed more diabetes than TLR5-sufficient NOD mice at the same age (**Figure 6D**). This may suggest that prolonged immune dysfunction is required to reach sufficient thresholds to accelerate diabetes development. This may also suggest that TLR5 has a stronger effect on the progression, rather than the initiation, of diabetes development, which may prove to be important therapeutically.

## 4 Discussion

In this study, we investigated the role of TLR5-deficiency in modulating phenotypes and functions of immune cells in an autoimmune diabetes-susceptible mouse model, and identified a number of novel findings. Firstly, we identified that TLR5-deficiency strongly influences DC development in mice in the prenatal period prior to microbial colonization and the changes were sustained into adulthood, including changes in CD103^+^ CD8α^-^ and CD103^+^CD8α^+^ DC subsets. Secondly, in the absence of TLR5, DCs were more responsive to both innate and adaptive immune stimuli. Thirdly, in the absence of TLR5, the NOD mice had altered gut microbiota; and direct exposure to the altered gut microbiota led to enhanced TNFα responses by DCs. Interestingly, direct exposure to the altered gut microbiota induced hyper cytokine responses from macrophages and T cells regardless of TLR5 expression. Fourthly, TLR5-deficient DCs enhance CD4^+^ T cell proliferation and cytokine secretion in responses to both polyclonal TCR activation and antigen-specific TCR engagement. Finally, and unexpectedly, TLR5-deficiency only affected spontaneous diabetes development in older NOD mice.

DCs are potent antigen-presenting cells that are vital for mediating insulitis and β-cell destruction (35, 36, 47, 48). DCs are also hetergeneous, with CD8α and CD103 commonly identifying lymphoid and migratory (or tissue resident) DCs, respectively (49–51). Fujimoto and coauthors reported that, in mouse lamina propria CD103^+^CD8α^+^ DCs expressed TLR3, TLR7 and TLR9, whereas CD103^+^CD8α^-^ DCs expressed TLR5 and TLR9 (3). As we observed reduced CD103^+^CD8α^+^ DCs (which express TLR3) but increased CD103^+^CD8α^-^ DCs (**Figures 2E–G**) in the TLR5 deficient mice, this may explain the reduced proliferation to PolyIC (TLR3 agonist), while no differences were seen in response to LPS or Pam3Csk4. The reduction in the CD103^+^CD8α^+^ DC subset that we observed in TLR5-deficient NOD mice may suggest that this subset of cells requires TLR5 signalling for development. Furthermore, in TLR5-deficient DCs, we identified that the gene expression of *Baft3* and *Id2*, transcription factors important in regulating differentiation of DC subsets (45, 46), were reduced. Interestingly, Id2-deficient mice have severely reduced proportions of CD8α^+^ DCs (45), while Baft3-deficient mice have reductions in both CD103^+^ and CD8α^+^ DCs subsets (37, 46), which suggest an explanation for the reduction of CD103^+^CD8α^+^ DCs observed in the TLR5-deficient mice. The altered DC subsets were important in modulating CD4^+^ T cell function. Interestingly, a previous study investigating DCs in the pancreatic islets of NOD mice, revealed that CD103^+^ DCs increased in abundance coinciding with CD4^+^ T cells infiltrating into the islets (37), thus confirming that interactions between CD103^+^ DCs and CD4^+^ T cells are important for mediating autoimmune diabetes development.

CD103^+^ DCs can sample microbial antigens from the intestinal lumen (31, 33); however, our results from the direct interaction of DCs with the microbiota does not support the notion that the microbiota are the cause of the altered DC function seen in TLR5-deficient mice. It is possible that other TLRs are involved, as many TLRs, except TLR2, are upregulated in the intestine of TLR5-deficient mice. Genes related to LPS signalling, including TLR4, are increased in the colon of TLR5-deficient mice (16). As TLR5-deficiency can alter microbial composition (16, 17), bacterial ligands and metabolites can also travel through the placenta, it is possible that maternally-derived microbial components from the TLR5-deficient parent can alter the TLR expression of the mice *in utero*. DCs in the human fetus can respond to TLR ligands (52), which may suggest their function is being altered early on in TLR5-deficient mice. Thus, the altered microbiota caused by TLR5 deficiency can alter the signaling through other TLRs. It is also possible that the microbe-derived products e.g. short-chain fatty acids (SCFAs), rather then the bacterial cells, modulate the DC function. Previous studies have shown that SCFAs can modulate DC maturation and functions (53–55). In the prenatal TLR5-deficient mice, we observed increased gene expression of common SCFA receptors (Gpr41/43) in the intestinal tissue prior to detectable evidence of microbial colonization. Interestingly, a study in humans has shown that TLR5 can bind butyrate, a SCFA, altering immune responses (56). This suggests that the maternal SCFAs may play a role in the altered DCs in the prenatal TLR5-deficient mice, the phenotype of which is still observed in adulthood. Furthermore, a study comparing the fetal intestine from germ-free and specific pathogen-free mice showed that microbiota have a significant influence on fetal metabolome, including tryptophan metabolites (57). Kynurenine, a tryptophan-derived metabolite, has been showed to significantly alter DC function (58), thus, it is likely that TLR5-deficiency alters microbial derived ligands and/or metabolites, both directly and indirectly, that drive changes of immune functions, including those observed in the DCs.

In all immune cells investigated, we observed increased TNFα secretion, either *ex vivo* or after *in vitro* stimulation, especially when TLR5-deficient DCs were present. Interestingly, TNFα is increased in the serum of patients with type 1 diabetes (59) and both Etanercept (a recombinant soluble TNFα receptor fusion protein, that binds TNFα) and Golimumab (anti-TNFα antibody) has been shown to lower HbA1c levels and promote insulin production in children/young adults with new-onset T1D (60, 61). It is conceivable that altered TLR5 signaling may play a role in the increased TNFα levels in patients with type 1 diabetes. As our data indicate, the acceleration in type 1 diabetes was observed only in older mice, over 17-weeks of age. Thus, understanding the impact of age in responses to TNFα-targeting therapies should be performed, as well as the role of TLR5 in modulating TNFα secretion and inflammatory dendritic cell development. Furthermore, flagellin, a TLR5 agonist, can act as an adjuvant for influenza vaccination to promote the development of protective vaccine-induced immunity in the elderly, through increased seroconversion and antibody titers (62). Furthermore, TLR5 expression increased post-influenza vaccination in PBMCs from vaccinees and strongly correlated with vaccine-induced antibody responses (63). Thus, given that TLR5 can modulate DC development and function, flagellin may be a useful agonist for promoting protective over inflammatory immune responses.

Despite the hyper DC responses accompanied by increased secretion of various cytokines in the absence of TLR5, in both prenatal and adult TLR5-deficient mice, autoimmune diabetes development was only increased in older adult mice (older than 17-weeks of age). DCs control both T cell entry and islet antigen presentation within the islets and are vital for regulating T cell-mediated β-cell destruction (35–37, 47, 48). As we observed reduced DC populations in both neonatal and adult mice, it is plausible that the delay in modulating diabetes susceptibility is related to fewer DCs enabling T cell entry or antigen presentation within the islets.

In summary, our data suggest that TLR5 signaling is important for DC development and maturation at the prenatal stage and maintained into adulthood. We found that TLR5-deficient DCs are hyper cytokine secretors that can modulate antigen-specific CD4^+^ T cell proliferation, leading to increased diabetes development in older adult mice. Our data suggest TLR5 signaling is important and thus suggest that TLR5 agonists (e.g. flagellin), may be a useful addition to immunotherapy in older individuals with T1D.

## Data availability statement

The datasets presented in this study can be found in online repositories. The names of the repository/repositories and accession number(s) can be found below: https://www.ncbi.nlm.nih.gov/, PRJNA1046460.

## Ethics statement

The animal study was approved by The Institutional Animal Care and Use Committee at Yale University. The study was conducted in accordance with the local legislation and institutional requirements.

## Author contributions

JAP: Data curation, Formal analysis, Funding acquisition, Investigation, Methodology, Project administration, Resources, Supervision, Validation, Visualization, Writing – original draft, Writing – review & editing. YH: Data curation, Formal analysis, Investigation, Supervision, Writing – original draft, Writing – review & editing. JP: Data curation, Formal analysis, Investigation, Methodology, Writing – original draft, Writing – review & editing. FW: Supervision, Writing – original draft, Writing – review & editing. LW: Conceptualization, Formal analysis, Funding acquisition, Methodology, Resources, Supervision, Validation, Writing – original draft, Writing – review & editing.
