# Supplementary material for: TLR5-deficiency controls dendritic cell subset development in an autoimmune diabetes-susceptible model
Source: Front Immunol. 2024 Feb 28;15:1333967. doi: 10.3389/fimmu.2024.1333967 (PMC10935730; doi:10.3389/fimmu.2024.1333967)
Supplement: Supplementary file 1 [file DataSheet_1.docx]

Supplementary Material

**TLR5-deficiency Controls Dendritic Cell Subset Development in an Autoimmune Diabetes-susceptible Model**

**James Alexander Pearson^1,2*^, Youjia Hu^1^, Jian Peng^1^, F. Susan Wong^2^ & Li Wen^1*^**

^1^ Section of Endocrinology, School of Medicine, Yale University, New Haven, Connecticut, USA

^2^ Diabetes Research Group, Division of Infection and Immunity, School of Medicine, Cardiff University, Cardiff, UK

*** Correspondence:**Dr James Alexander Pearson or Professor Li Wen
[pearsonj1@cardiff.ac.uk](mailto:pearsonj1@cardiff.ac.uk) or [li.wen@yale.edu](mailto:li.wen@yale.edu)

# Supplementary Figures and Tables

## Supplementary Figures


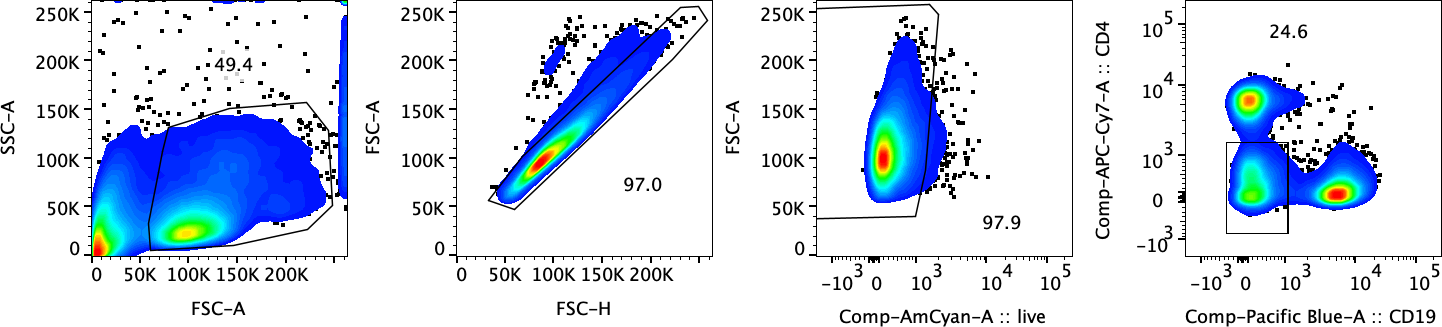

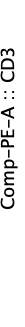


**Supplementary Figure 1 – DC gating of mice studied *in utero.*** Splenocytes from E17 mice *in utero* were harvested from TLR5-sufficient (WT) or TLR5-deficient (KO) pregnant females, 17-days post-plug formation. A representative example of gating is shown using SSC-A vs FSC-A to identify the cells, prior to gating on single cells (FSC-A vs FSC-H), then live cells (FSC-A vs Live/Dead viability dye (AmCyan)), then CD3 and CD19 negative cells. DCs and macrophages were subsequently gated as in Fig 1A.

CD11c

TNF$\alpha$

CD11c

IL-10

CD11c

IL-4

CD11c

IFN$\gamma$

**WT**

**KO**

**Isotype**


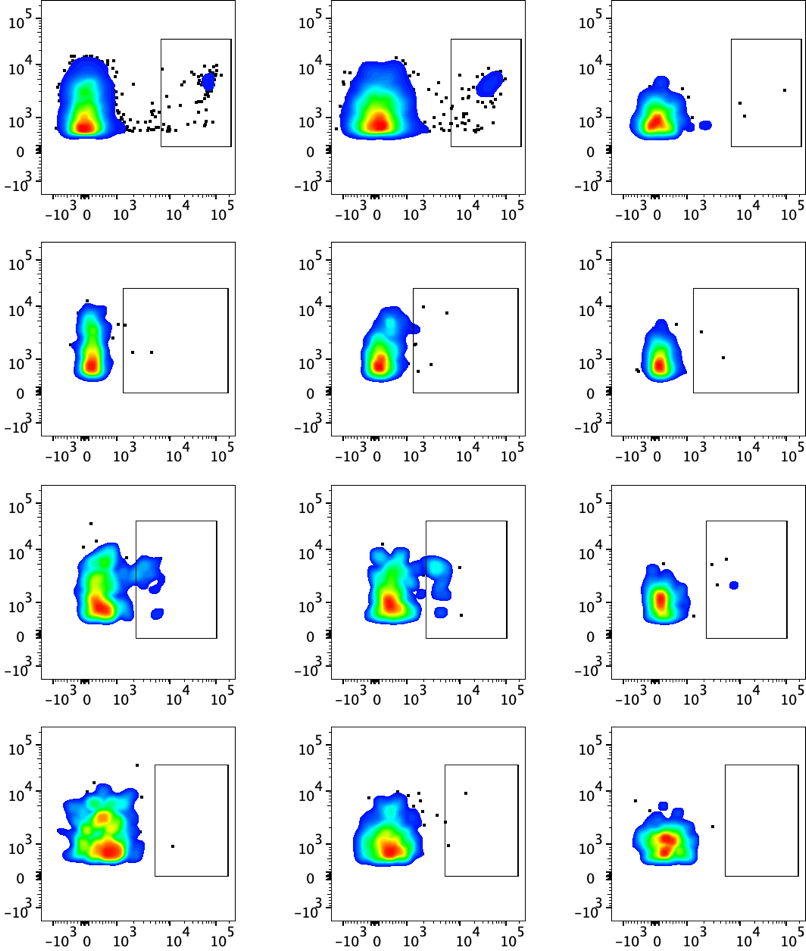


0.220

0.070

0

8.310

4.548

0.670

0.540

0.280

0.210

8.700

4.060

0.330

**Supplementary Figure 2 – Representative gating of cytokine-secreting CD11c^+^CD11b^-^ cells.** Splenocytes from E17 mice *in utero* were harvested from TLR5-sufficient (WT) or TLR5-deficient (KO) pregnant females, 17-days post-plug formation, prior to cell surface and intracellular staining. Representative flow cytometric plots gated on single, live CD19^-^CD3^-^CD11c^+^CD11b^-^ cells prior to gating on TNFα^+^, IL-10^+^, IL-4^+^ or IFNγ^+^ populations using isotype controls.

CD11b

TNF$\alpha$

CD11b

IL-10

CD11b

IL-4

CD11b

IFN$\gamma$

**WT**

**KO**

**Isotype**


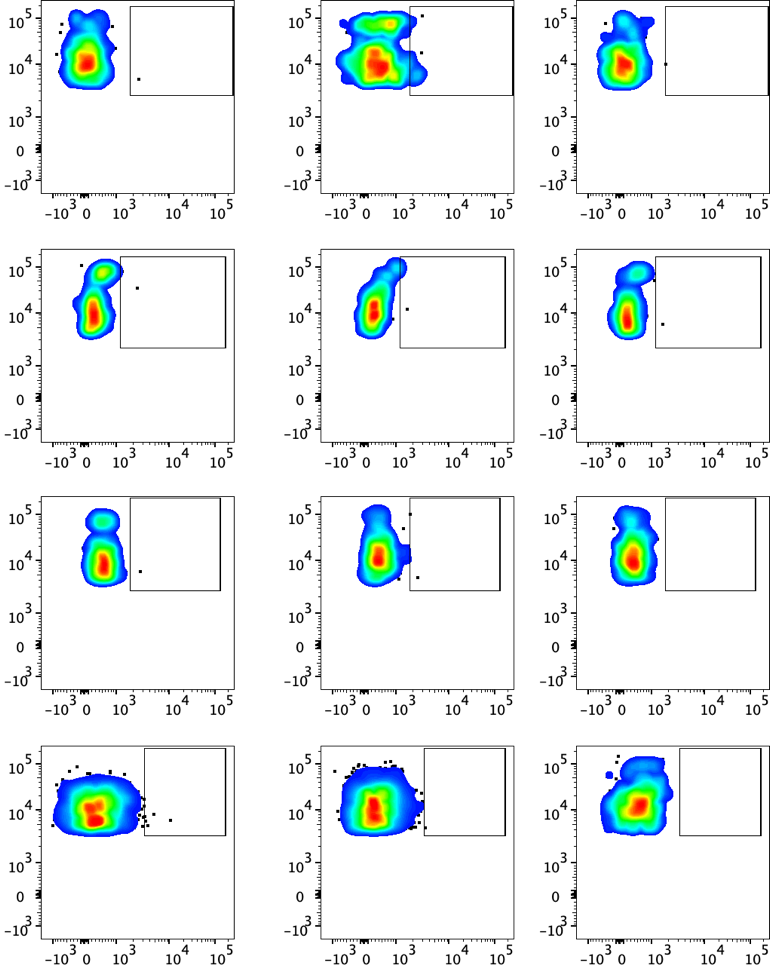


0.230

0.100

0

0.390

0.610

0

0.190

0.320

0.150

1.150

0.150

0.090

**Supplementary Figure 3 – Representative gating of cytokine-secreting CD11c^+^CD11b^+^ cells.** Splenocytes from E17 mice *in utero* were harvested from TLR5-sufficient (WT) or TLR5-deficient (KO) pregnant females, 17-days post-plug formation, prior to cell surface and intracellular staining. Representative flow cytometric plots gated on single, live CD19^-^CD3^-^CD11c^+^CD11b^+^ cells prior to gating on TNFα^+^, IL-10^+^, IL-4^+^ or IFNγ^+^ populations using isotype controls.

CD11b

TNF$\alpha$

CD11b

IL-10

CD11b

IL-4

CD11b

IFN$\gamma$


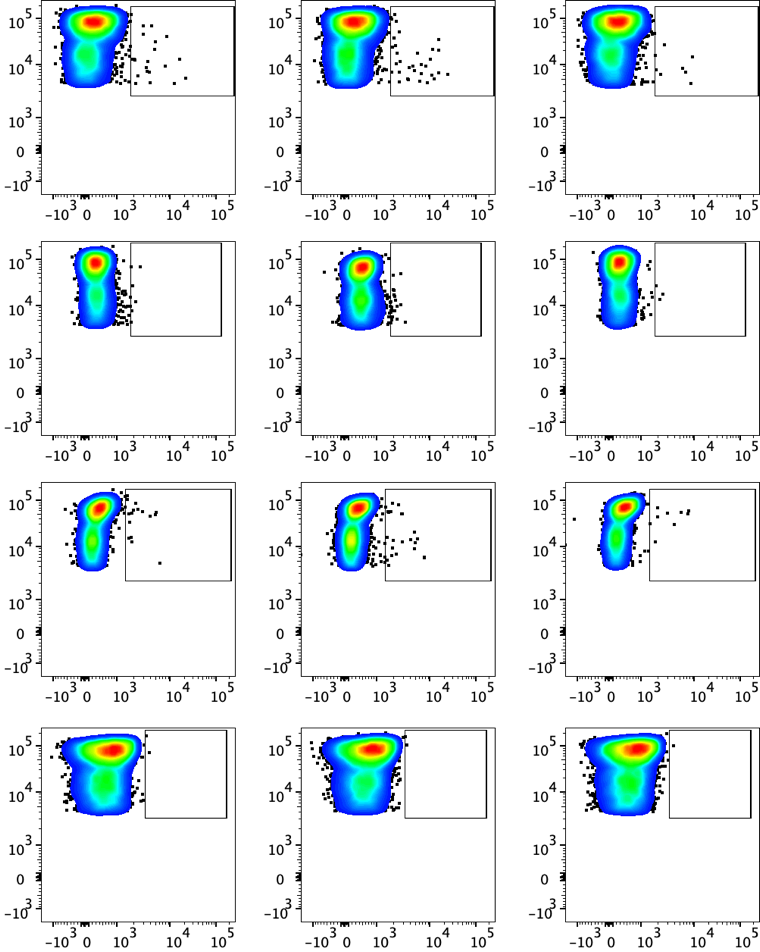


**WT**

**KO**

**Isotype**

0.010

0.012

0.007

0.670

0.270

0.180

0.026

0.083

0.021

0.183

0.310

0.560

**Supplementary Figure 4 – Representative gating of cytokine-secreting CD11b^+^CD11c^-^ cells.** Splenocytes from E17 mice *in utero* were harvested from TLR5-sufficient (WT) or TLR5-deficient (KO) pregnant females, 17-days post-plug formation, prior to cell surface and intracellular staining. Representative flow cytometric plots gated on single, live CD19^-^CD3^-^CD11b^+^CD11c^-^ cells prior to gating on TNFα^+^, IL-10^+^, IL-4^+^ or IFNγ^+^ populations using isotype controls.

A

B

C

D

**Supplementary Figure 5 – TLR5-deficiency does not alter frequency of B or T cells *in utero.*** Splenocytes and thymocytes from E17 mice *in utero* were harvested from TLR5-sufficient (WT) or TLR5-deficient (KO) pregnant females, followed by cell surface (A, C-D) or intracellular staining (B). **(A**) Proportion of splenic B cells gated from single, live CD19^+^TCRβ^-^ cells. **(B**) Cytokine-producing splenic B cells, gated as the cells in (A), prior to gating on IFNγ^+^, IL-4^+^, IL-10^+^ or TNFα^+^ populations. (**C**) Thymic T cell proportions, gated from live, single cells prior to CD4 and CD8 gating. (**D**) Splenic T cell proportions, gated from live, single CD19^-^TCRβ^+^ cells prior to CD4^+^ and CD8^+^ gating. Data shown are representative of 1 of 2 independent experiments (A-D: n=4-8/experiment) and were assessed for significance using a Student’s T Test.

**
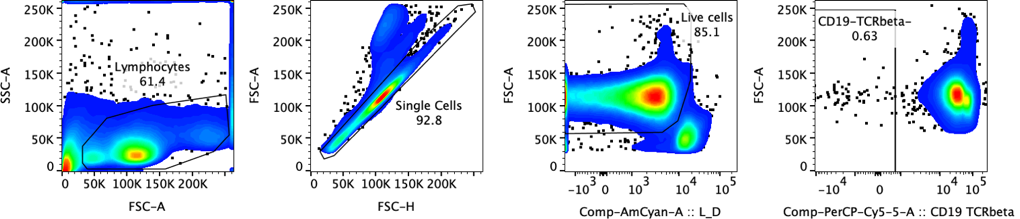
**

**Supplementary Figure 6 – DC gating of 8-week-old mice*.*** DC subsets from 8-week-old TLR5-sufficient (WT) or TLR5-deficient (KO) mice were studied. A representative example of gating is shown using SSC-A vs FSC-A to identify the cells, prior to gating on single cells (FSC-A vs FSC-H), then live cells (FSC-A vs Live/Dead viability dye (AmCyan)), then TCR$\beta$ and CD19 negative cells (both stained on cells using antibodies conjugated to PerCpCy5.5). DCs and macrophages were subsequently gated as in Fig 2A.

A

B

C

D

E

F

G

H

I

J

**Supplementary Figure 7 – TLR5-deficiency minimally alters other APC subsets in adult mice.** (**A-B**) The proportion of CD11b^+^CD11c^+^CD103^+^CD8α^-^ (A) or CD11b^+^CD11c^+^CD103^+^CD8α^+^ DCs (B) were investigated from the spleen, pancreatic lymph nodes (PLN), mesenteric lymph nodes (MLN) and Peyer’s patches (PP) of 8-week-old TLR5-sufficient (WT) or TLR5-deficient (KO) mice. Cells were gated from live, single I-Ag7^+^CD19^-^TCRβ^-^CD11b^+^CD11c^+^ cells prior to gating on CD103 and CD8α. (**C-D**) The proportion of CD11b^+^CD11c^-^CD103^+^CD8α^-^ (C) or CD11b^+^CD11c^-^CD103^+^CD8α^+^ macrophages (D) gated from live, single CD19^-^TCRβ^-^CD11c^-^ cells prior to gating on CD103 and CD8α. (**E-G**) The proportion of cytokine-secreting CD11b^+^CD11c^+^ (E), CD11b^+^CD11c^-^ (F) or CD19^+^ (G) cells were investigated by flow cytometry from the PLN. Cells were gated as above (for CD11b^+^CD11c^+^ or CD11b^+^CD11c^-^ cells or from live, single TCRβ^-^CD19^+^ cells for B cells) prior to gating on IFNγ^+^, IL-4^+^, IL-6^+^, IL-10^+^, TGFβ^+^ or TNFα^+^ populations. (**H-I**) The proportion of CD138^+^ plasma B cells (H) and IgA^+^ B cells (I). (**J**) The proportion of cytokine-secreting CD8^+^ T cells were investigated by flow cytometry from the PLN. Cells were gated from live, single TCRβ^+^CD8^+^CD4^-^CD19^-^ cells prior to gating on Granzyme B^+^, IFNγ^+^, IL-10^+^ or TNFα^+^ populations. Data shown are pooled from 2 independent experiments (A-J: n=6, n=3/experiment) and were assessed for significance using a Student’s T Test.


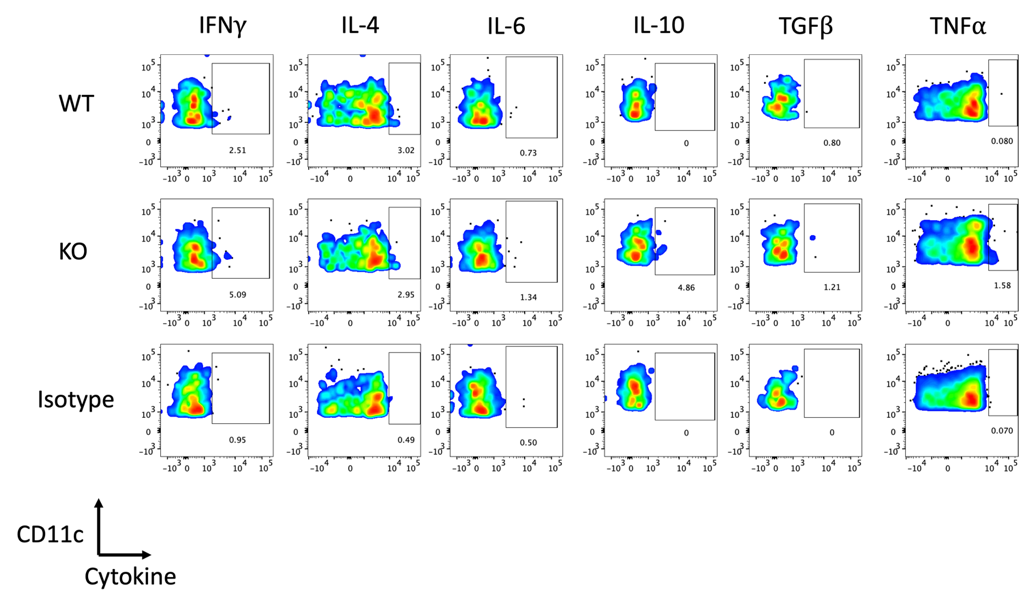


**Supplementary Figure 8 – Representative gating of intracellular cytokine secretion from CD11c+CD11b- DCs.** Cytokine-secreting DCs (live, single CD19^-^TCRβ^-^CD11c^+^CD11b^-^) from 8-week-old TLR5-sufficient (WT) or TLR5-deficient (KO) mice were studied 4-hours post-PMA and Ionomycin stimulation in the presence of GolgiPlug. Representative gating of IFNγ^+^, IL-4^+^, IL-6^+^, IL-10^+^, TGFβ^+^ or TNFα^+^ populations are shown with the respective isotype control.

**
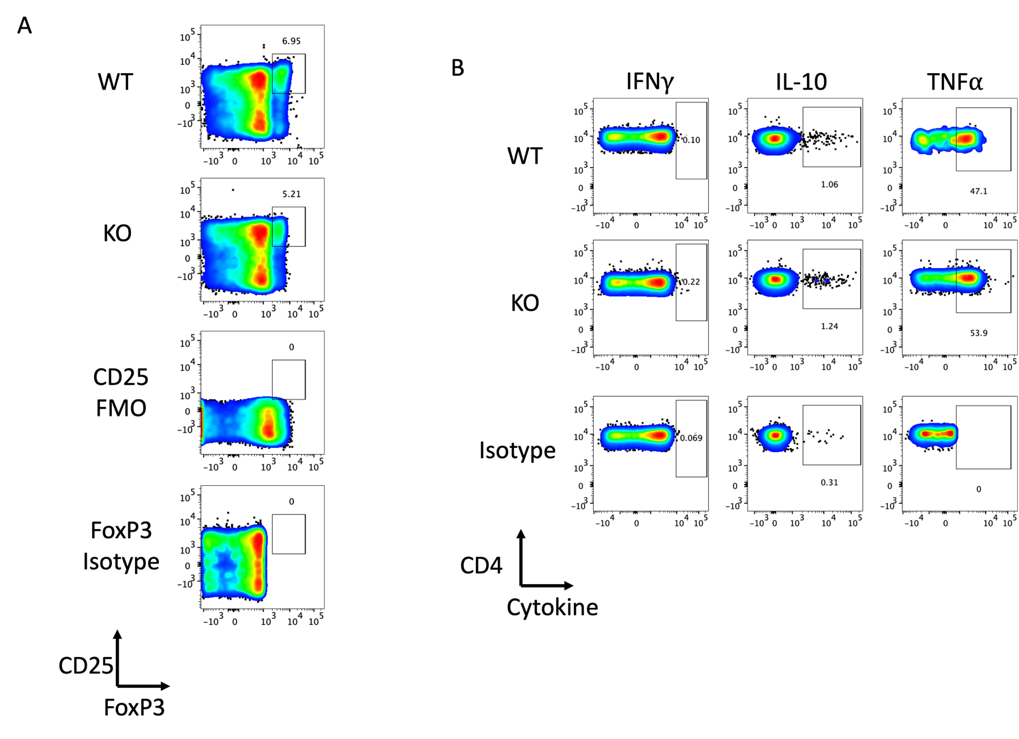
**

**Supplementary Figure 9 – Representative gating of Tregs and cytokine-secreting CD4^+^ T cells.** (**A**) Representative gating of Tregs, gated from live, single TCRβ^+^CD4^+^CD19^-^CD8^-^ cells, prior to gating on CD25^+^FoxP3^+^ cells using CD25 fluorescent minus one (FMO) or FoxP3 isotype controls. (**B**) Representative gating of cytokine-secreting CD4^+^ T cells 4-hours post-PMA and Ionomycin stimulation in the presence of GolgiPlug. Live, single TCRβ^+^CD4^+^CD19^-^CD8^-^ cells were gated, prior to gating on IFNγ^+^, IL-10^+^ or TNFα^+^ populations using isotype controls.

B

C

D

**LPS**

**Pam3Csk4**

A

**Supplementary Figure 10 – TLR5-deficiency alters DC cytokine responses to LPS and Pam3Csk4.** CD11c^+^ DCs were purified from the spleen of 8-week-old TLR5-sufficient (WT) or TLR5-deficient (KO) mice and stimulated for 48 hours in the presence of various concentrations of LPS (A; TLR4 agonist) or Pam3Csk4 (B; TLR2 agonist), prior to supernatant collection and the addition of ^3^H-thymidine. Cells were incubated for a further 18 hours prior to harvesting and analysis on a β-counter. (**C-D**) Cell supernatants of (A-B) were investigated for cytokine concentrations by ELISA. Data shown are from the highest concentration of stimulation. Data are plotted as counts per minute corrected for background (no stimulation (ΔCPM)) or cytokine concentration corrected for background (no stimulation (Δ Cytokine (pg/ml))). Data are averaged from triplicates or duplicates respectively. Data shown are representative of 1 of 3 independent experiments (A-D: n=3-4/experiment) and were assessed for significance using a Mann-Whitney test.

A


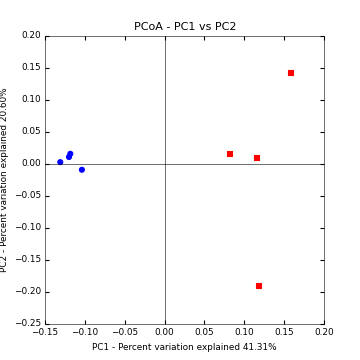


WT NOD

KO NOD

PC1 – Percent variation (41.31%)

PC2 – Percent variation (20.60%)

D

B

C

E

**Supplementary Figure 11 – TLR5-deficiency alters the gut microbiota composition.** Fecal pellets were collected from 8-week old TLR5-sufficient (WT) or TLR5-deficient (KO) mice. Bacterial DNA was extracted and subjected to 16S rRNA deep sequencing.  **(A**) Principal component analysis of β-diversity of the gut microbiota. (**B-E**) Microbial species contributing to the altered β-diversity. *Lactobacillus reuteri* (B; Firmicutes phylum member), *Mycoplasma muris* (C; Tenericutes phylum member), *Rickenellaceae* and *Bacteroidales* species (D-E; Bacteroidetes phyla members). *Rickenellaceae* species refers taxonomically to *Rickenellaceae* family members with an unknown genus and species. *Bacteroidales* species refers taxonomically to *Bacteroidales* order members with an unknown family, genus and species. Data shown are from 1 experiment (A-E: n=4) and were assessed for significance using an ANOSIM (A) or Student’s T Test (B-E).

**
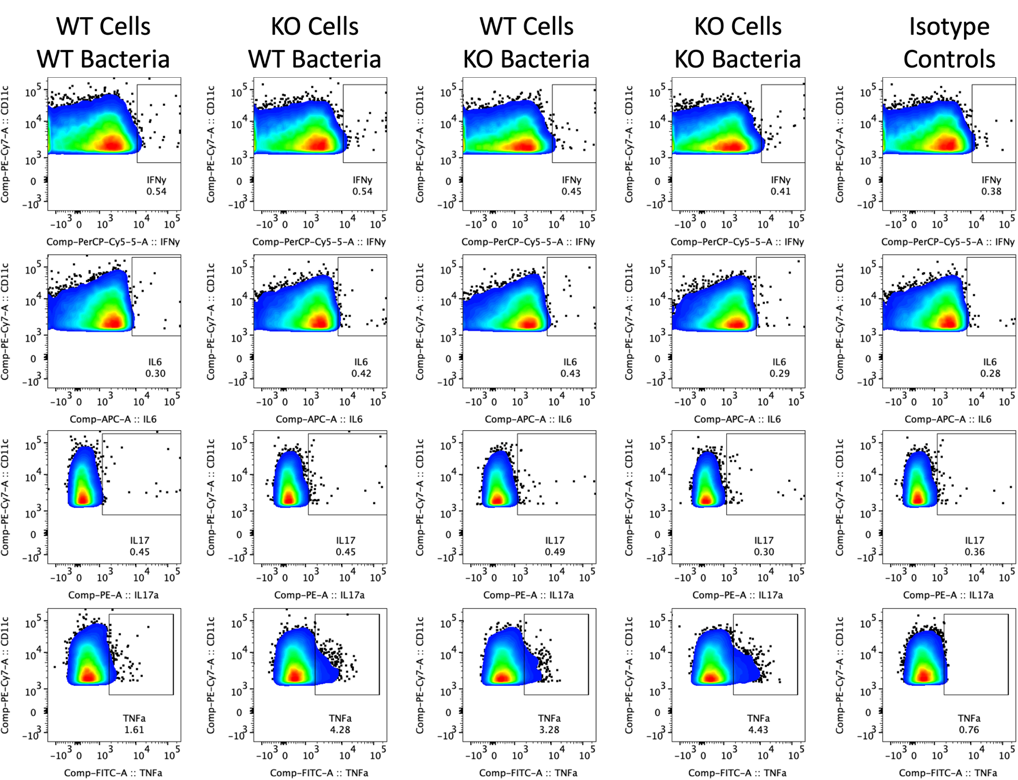
**

**Supplementary Figure 12 – Representative gating of microbiota-stimulated CD11c^+^CD11b^-^ cells.** 12-hours post-heat-inactivated fecal microbiota stimulation of splenocytes from TLR5-sufficient (WT) and TLR5-deficient (KO), cells were stimulated for 4 hours with PMA and Ionomycin in the presence of GolgiPlug. Representative gating of IFNγ^+^, IL-6^+^, IL-17A^+^ and TNFα^+^ CD11c^+^ DCs are shown with isotype controls.


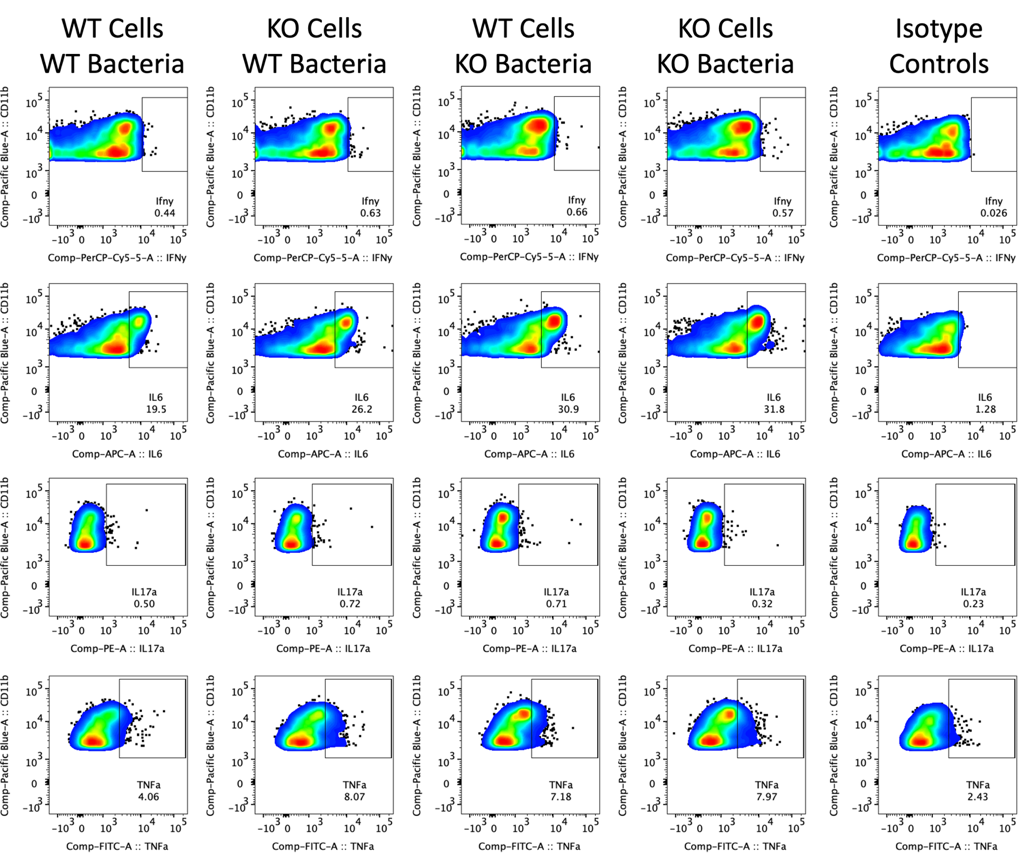


**Supplementary Figure 13 – Representative gating of microbiota-stimulated CD11b^+^CD11c^-^ cells.** 12-hours post-heat-inactivated fecal microbiota stimulation of splenocytes from TLR5-sufficient (WT) and TLR5-deficient (KO), cells were stimulated for 4 hours with PMA and Ionomycin in the presence of GolgiPlug. Representative gating of IFNγ^+^, IL-6^+^, IL-17A^+^ and TNFα^+^ CD11b^+^ cells are shown with isotype controls.

**
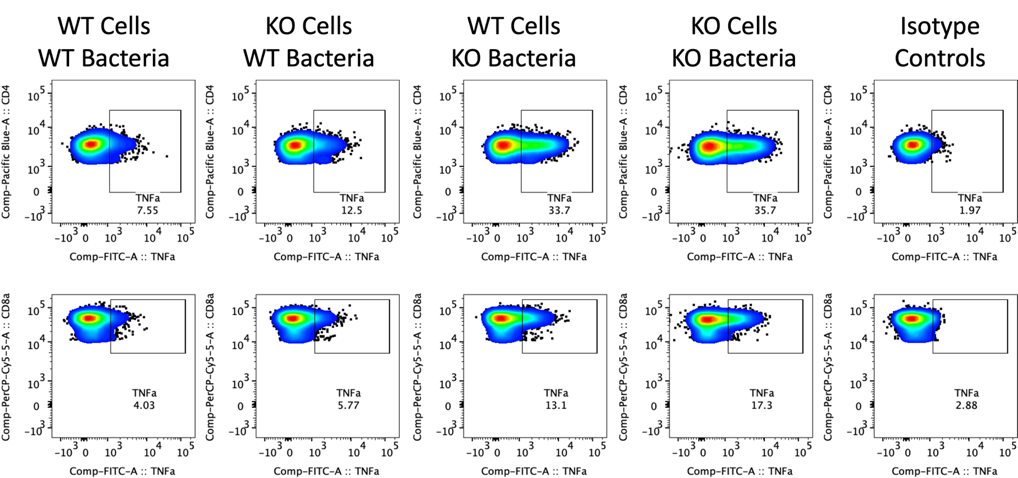
**

**Supplementary Figure 14 – Representative gating of microbiota-stimulated CD11b^+^CD11c^-^ cells.** 12-hours post-heat-inactivated fecal microbiota stimulation of splenocytes from TLR5-sufficient (WT) and TLR5-deficient (KO), cells were stimulated for 4 hours with PMA and Ionomycin in the presence of GolgiPlug. Representative gating of TNFα^+^ CD4^+^ and CD8^+^ T cells (top and bottom row respectively) are shown with isotype controls.

B

A

**Supplementary Figure 15 – TLR5-deficient DCs do not influence antigen-specific CD8^+^ T cell proliferation or TNF**$\boldsymbol{\alpha}$ **secretion.** CD11c^+^ DCs were purified from 8-week-old TLR5-sufficient (WT) and TLR5-deficient (KO) mice. CD8^+^ T cells were purified from NY8.3 T cell receptor transgenic mice. NY8.3 CD8^+^ T cells (1x10^5^/well) and mitomycin-c-treated CD11c^+^ DCs (1x10^5^/well) were cultured 1:1 in the presence of various concentrations of IGRP peptide for 48 hours prior to supernatant collection and ^3^H-Thymidine addition. Cells were cultured for a further 18 hours prior to harvesting. ^3^H-Thymidine incorporation proliferation of NY8.3 CD8^+^ T cells in the presence of WT or KO CD11c^+^ was analyzed on a β-counter. Secreted TNFα was measured from the supernatant in (A). Data shown are pooled from 3 independent experiments (n=9, n=3/experiment). A Two-way ANOVA was used to assess significance.


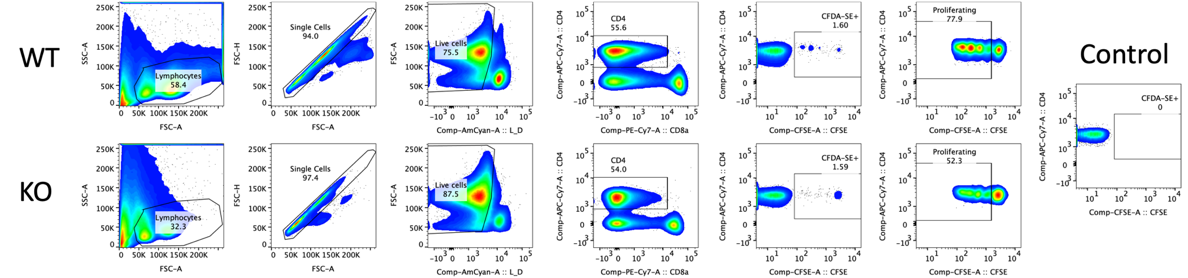


**Supplementary Figure 16 – Representative gating of microbiota-stimulated CD11b^+^CD11c^-^ cells.** CFSE-labelled BDC2.5 CD4^+^ T cells (2x10^6^) were adoptively transferred into WT or KO mice and assessed for proliferation after 3 days in vivo. Representative gating is shown using SSC-A vs FSC-A to identify the cells, prior to gating on single cells (FSC-A vs FSC-H), then live cells (FSC-A vs Live/Dead viability dye (AmCyan)), then CD4^+^ cells, then CFSE^+^ cells, prior to proliferating cells (diluted CFSE). A control mouse that did not receive CFSE^+^ cells was used as a control for gating.

**Supplementary Table 1 – qPCR primer sequences**

| **Gene name** | **Forward primer (5’-3’)** | **Reverse primer (5’-3’)** |
| --- | --- | --- |
| *tlr1* | ACTTGCCTTCAGGATGTTCAAT | TAAAGTCCACATGCAGAAATGG |
| *tlr2* | GCTCAGGAGTCTCTGTCATGTG | GGCGTCTCCCTCTATTGTATTG |
| *tlr3* | GTGAGATACAACGTAGCTGACTG | TCCTGCCATCCAAGATAGCAAGT |
| *tlr4* | TGTCATCAGGGACTTTGCTG | GGACTCTGATCATGGCACTG |
| *tlr5* | ATTCCTCGTCATCACCCTTG | GCATAGCCTGAGCCTGTTTC |
| *tlr6* | AACTGACCTTCCTGGGTGTG | CATTGGAATGGGTTGTTTCC |
| *tlr7* | ATGTGGACACGGAAGAGACAA | GGTAAGGGTAAGATTGGTGGTG |
| *tlr8* | ACAATGCTCCATTTCCTTGC | CTGAGGGAAGTGCTGGAAAG |
| *tlr9* | ACGGGAACTGCTACTACAAGA | CCCAGCTTGACAATGAGGTTAT |
| *gpr41* | GTGACCATGGGGACAAGCTTC | CCCTGGCTGTAGGTTGCATT |
| *gpr43* | GGCTTCTACAGCAGCATCTA | AAGCACACCAGGAAATTAAG |
| *reg3β* | CTGCCTTAGACCGTGCTTTC | CCCTTGTCCATGATGCTCTT |
| *reg3γ* | TTCCTGTCCTCCATGATCAAAA | CATCCACCTCTGTTGGGTTCA |
| *β-defensin 14* | AAGTACAGCACACCGGCCAC | GTATTCCTCATCTTGTTCTTGG |
| *defcr* | ATCATCCAGGTGATTCCCAGCCAT | TTCCGGGTCTCCAAAGGAAACAGA |
| *crp-ductin* | TGAACCGTGTGACAGTGGTCTTCA | TCTCCTTGTCACACTGCCATCTGT |
| *relmb* | AGCTCTCAGTCGTCAAGAGCCTAA | CACAAGCACATCCAGTGACAACCA |
| *baft3* | CTGCGCAGCACAGAGTTCTC | CAGACCCAGAAGGCTGACAAG |
| *id2* | AACGTGTTCTCCTGGTGAAATGGC | TCCTGTCCTTGCAGGCATCTGAAT |
| *flt3* | TCCAAGGGCGGGTGTAACTGAACT | GTGACTGGCCCCCTGGATAACGAG |
| *zbtb46* | CTGGCTGCAGACATGAACAC | AGAGAGCACATGAAGCGACA |
| *gapdh* | TGACATCAAGAAGGTGGTGAAG | TGCTGTAGCCGTATTCATTGTC |
